# Sepsis Prediction Model for Determining Sepsis vs SIRS, qSOFA, and SOFA

**DOI:** 10.1001/jamanetworkopen.2023.29729

**Published:** 2023-08-25

**Authors:** Adam R. Schertz, Kristin M. Lenoir, Alain G. Bertoni, Beverly J. Levine, Morgana Mongraw-Chaffin, Karl W. Thomas

**Affiliations:** 1 Department of Internal Medicine, Atrium Health Wake Forest Baptist, Winston-Salem, North Carolina; 2 Section of Pulmonology, Critical Care, Allergy and Immunologic Diseases, Atrium Health Wake Forest Baptist, Winston-Salem, North Carolina; 3 Department of Biostatistics and Data Science, Division of Public Health Science, Atrium Health Wake Forest Baptist, Winston-Salem, North Carolina; 4 Social Sciences and Health Policy, Wake Forest University School of Medicine, Winston-Salem, North Carolina; 5 Department of Epidemiology and Prevention, Atrium Health Wake Forest Baptist Winston-Salem, North Carolina

## Abstract

**Importance:**

The Sepsis Prediction Model (SPM) is a proprietary decision support tool created by Epic Systems; it generates a predicting sepsis score (PSS). The model has not undergone validation against existing sepsis prediction tools, such as Systemic Inflammatory Response Syndrome (SIRS), Sequential Organ Failure Assessment (SOFA), or quick Sepsis-Related Organ Failure Asessement (qSOFA).

**Objective:**

To assess the validity and timeliness of the SPM compared with SIRS, qSOFA, and SOFA.

**Design, Setting, and Participants:**

This retrospective cohort study included all adults admitted to 5 acute care hospitals in a single US health system between June 5, 2019, and December 31, 2020. Data analysis was conducted from March 2021 to February 2023.

**Main Outcomes and Measures:**

A sepsis event was defined as receipt of 4 or more days of antimicrobials, blood cultures collected within ±48 hours of initial antimicrobial, and at least 1 organ dysfunction as defined by the organ dysfunction criteria optimized for the electronic health record (eSOFA). Time zero was defined as 15 minutes prior to qualifying antimicrobial or blood culture order.

**Results:**

Of 60 507 total admissions, 1663 (2.7%) met sepsis criteria, with 1324 electronic health record–confirmed sepsis (699 [52.8%] male patients; 298 [22.5%] Black patients; 46 [3.5%] Hispanic/Latinx patients; 945 [71.4%] White patients), 339 COVID-19 sepsis (183 [54.0%] male patients; 98 [28.9%] Black patients; 36 [10.6%] Hispanic/Latinx patients; and 189 [55.8%] White patients), and 58 844 (97.3%; 26 632 [45.2%] male patients; 12 698 [21.6%] Black patients; 3367 [5.7%] Hispanic/Latinx patients; 40 491 White patients) did not meet sepsis criteria. The median (IQR) age was 63 (51 to 73) years for electronic health record–confirmed sepsis, 69 (60 to 77) years for COVID-19 sepsis, and 60 (42 to 72) years for nonsepsis admissions. Within the vendor recommended threshold PSS range of 5 to 8, PSS of 8 or greater had the highest balanced accuracy for classifying a sepsis admission at 0.79 (95% CI, 0.78 to 0.80). Change in SOFA score of 2 or more had the highest sensitivity, at 0.97 (95% CI, 0.97 to 0.98). At a PSS of 8 or greater, median (IQR) time to score positivity from time zero was 68.00 (6.75 to 605.75) minutes. For SIRS, qSOFA, and SOFA, median (IQR) time to score positivity was 7.00 (−105.00 to 08.00) minutes, 74.00 (−22.25 to 599.25) minutes, and 28.00 (−108.50 to 134.00) minutes, respectively.

**Conclusions and Relevance:**

In this cohort study of hospital admissions, balanced accuracy of the SPM outperformed other models at higher threshold PSS; however, application of the SPM in a clinical setting was limited by poor timeliness as a sepsis screening tool as compared to SIRS and SOFA.

## Key Points

**Question:**

Does the Sepsis Prediction Model (SPM) outperform other sepsis prediction scores with respect to validity and timeliness?

**Findings:**

This cohort study of 60 507 adult admissions found that although balanced accuracy of the SPM at a predicting sepsis score (PSS) threshold of 8 or greater was better than that of the quick Sepsis-Related Organ Failure Assessment (qSOFA), Sequential Organ Failure Assessment (SOFA), and Systemic Inflammatory Response Syndrome (SIRS), there was longer time to score positivity from time zero for the SPM vs SIRS and SOFA.

**Meaning:**

While the balanced accuracy of the SPM was better than qSOFA, SOFA, and SIRS at higher-threshold PSS, it had poor timeliness for sepsis prediction.

## Teaser

This cohort study assesses the validity and timeliness of the Sepsis Prediction Model compared with the Systemic Inflammatory Response Syndrome (SIRS), Sequential Organ Failure Assessment (SOFA), and quick Sepsis-Related Organ Failure Assessment (qSOFA).

## Introduction

The Third International Task Force on Sepsis and Septic Shock (Sepsis-3) established a consensus definition of sepsis as “life-threatening organ dysfunction caused by a dysregulated host response to infection.”^1^ Although updated definitions have advanced the conceptual framework of sepsis, significant heterogeneity among patients with sepsis continues to limit the application of practical and efficient clinical tools to improve treatment delivery.^1,2,3,4,5,6,7^ This heterogeneity arises from the grouping of different infectious organisms, sites of infection, and organ dysfunctions into a single clinical condition rather than as unique disease states.^8^ At the point of care, no single marker or physiologic parameter consistently predicts the imminent development of sepsis. Additional challenges for sepsis recognition and intervention programs include prevention of harm through overtreatment with antibiotics and intravenous fluids.^9,10,11,12,13,14,15,16,17,18,19,20,21^ Consequently, early sepsis identification remains a major challenge with both overdiagnosis and underdiagnosis contributing to negative outcomes. Accurate, early recognition of sepsis linked to specific, early interventions is an important but elusive goal for developers of electronic decision support systems.^18,22,23,24,25,26,27,28^

Sepsis identification systems based on bedside findings are widely disseminated to promote early recognition and prompt intervention. Current recommendations emphasize that sepsis be considered a medical emergency and stress the importance of timely treatment.^29^ These goals of early recognition and intervention are impeded by suboptimal performance of simplified disease scales. For example, 2 common sepsis detection models are the Systemic Inflammatory Response Syndrome (SIRS) criteria, which has a pooled sensitivity of 88% and a specificity of 26%, and quick Sepsis-Related Organ Failure Assessment (qSOFA), which has a pooled sensitivity of 61% and a specificity of 72% for sepsis as defined by Sepsis-3 criteria.^1,30^ Newer, complex, and often proprietary models based on electronic health record (EHR) data including identification of organ dysfunction have been developed to improve the accuracy and timeliness of sepsis prediction.

The Sepsis Prediction Model (SPM; Epic Systems) is a proprietary algorithm developed from a pooled sample of 405 000 patient encounters across 3 health care organizations between 2013 and 2015.^31^ For model development, sepsis was defined as any encounter associated with an* International Classification of Disease, Ninth Revision* (*ICD-9*) code indicating diagnosis of sepsis. The SPM provides a predicting sepsis score (PSS), which is based on demographic, comorbidity, vital sign, laboratory, medication, and procedural variables. Variables included in the PSS are directly linked to previously established sepsis indicators, such as temperature, heart rate, respirations, and white blood cell count, as well as other clinical indicators of infection, such as orders for common antimicrobial classes. The PSS is recalculated every 15 minutes and can be linked to a threshold-based alert for medical staff to the risk for sepsis in an individual patient.^32^ A higher score indicates a higher likelihood the patient has sepsis. The SPM has not been independently validated in multiple different clinical environments and has not consistently demonstrated improved predictive scoring for sepsis compared with other models.^31,33,34^ This study aims to assess the validity and timeliness of the SPM for prediction of sepsis in a single health system with a large group of health care facilities and compare the performance of the Epic SPM to SIRS, qSOFA, and Sequential Organ Failure Assessment (SOFA).

## Methods

### Background

This is a retrospective cohort study of all adult (>17 years) admissions within 5 hospitals of Wake Forest Baptist Health (Winston-Salem, North Carolina) beginning June 5, 2019, through December 31, 2020. For the inpatient pool, a PSS was calculated every 15 minutes and recorded for all patients until discharge. This study was approved with a waiver of informed consent by the Wake Forest institutional review board because the study involved no more than minimal risk to participants. The study followed the Strengthening the Reporting of Observational Studies in Epidemiology (STROBE) reporting guidelines.^35^

### Study Population

Structured data from the EHR identified inpatient admissions and all associated characteristics reported in this study. We handled potential missing data exactly how an applied algorithm would handle these elements based on their presence in the EHR. Structured data elements (eg, temperature or blood pressure) that contributed to each criterion were collected heterogeneously, and inability to calculate a criterion within a prespecified time frame would not alert a clinician to elevated risk in the EHR and would therefore be considered a nonsepsis case at that time point. Structured data elements were reviewed for quality and plausibility based on clinician agreement, and clinically impossible values (eg, Glasgow Coma Scale of zero) were omitted from the calculation of each criterion and analyses. Race and ethnicity data were routinely collected in the EHR and are reported in this study. We observed low proportions of missingness for study data (eTable 1 in Supplement 1).

### Exclusion Criteria

For the analysis, we excluded admissions to a Burn Service, length of stay more than 30 days, those who left against medical advice, and transfers from an out-of-system facility or between internal sites. We excluded any sepsis-related readmissions within 30 days of an index admission for each patient due to the potential for related hospital visits that could affect patient condition or coding among subsequent visits. We excluded admissions for which there was no blood pressure or temperature recorded.

### Sepsis Definition

EHR-confirmed sepsis was defined by the Centers for Disease Control and Prevention (CDC) Adult Sepsis Event (ASE) criteria of (1) 4 or more days of qualifying antimicrobial therapy beginning within ±2 days of blood culture collection and (2) organ dysfunction as defined by organ dysfunction criteria optimized for EHR (eSOFA).^36^ Because the study timeframe was concurrent with the COVID-19 pandemic, a disease-specific definition of sepsis for COVID-19 was developed using the *International Statistical Classification of Diseases and Related Health Problems, Tenth Revision *(*ICD-10*) code of U07.1x^37^ and organ dysfunction during the admission in accordance with eSOFA criteria, but without the restriction of presence of antibiotic administration or blood culture. Septic shock was determined by an *ICD-10* code (R65.21, T81.12XA) or vasopressor use during a sepsis-related admission.^38^ The Charlson Comorbidity Index (CCI) was calculated by using historical *ICD-10* data collected within 2 years preceding the admission through the end of the admission. Comorbid conditions were reported from the components that contributed to the CCI calculation.^39^

### Electronic Sepsis Alert Systems

An electronic sepsis alert based on the SPM was active during all phases of care at a single site within the health system, with the alert threshold set to a PSS of 10 or greater. All other study sites had an electronic sepsis alert based on a positive SIRS score that was only active in the emergency department (ED). There was no mandatory action required on the part of health care practitioners for a positive sepsis screen at any study site.

### Time Zero

Time zero was set as 15 minutes prior to first clinician action, as defined by the initial order for antimicrobials or blood cultures. As stated previously, the SPM recalculates the PSS every 15 minutes. The calculation incorporates antimicrobial orders in the score derivation, causing score inflation of an unknown magnitude after an antimicrobial order. By assigning time zero as 15 minutes prior to clinician action, we allowed for a time-specific analysis of the SPM not directly influenced by clinician action related to suspicion or treatment of sepsis. For example, if a clinician assessed a patient and ordered antibiotics or a blood culture at 2:00 pm, time zero for that episode of care would be set at 1:45 pm.

The times elapsed for SIRS, qSOFA, and SOFA to reach threshold score were assessed at 1 hour. These intervals reflected the time from when any first criterion was present to the time when 2 or more criteria were present (SIRS, qSOFA) or to the time when an increase in score of 2 or greater (SOFA) was noted. We assessed timeliness relative to time zero from the point at which each of the criteria reached a positive threshold.

### Outcomes

Our primary goal was to compare the performance of the SPM in classifying sepsis admissions relative to SIRS, qSOFA, and SOFA, and examine the timeliness of each tool with respect to time zero. Performance was defined by comparison to the reference standard of EHR-confirmed sepsis using the CDC surveillance definition as previously described.

### Statistical Analysis

We performed statistical analysis using R Statistical Computing Environment version 4.0.5 (R Core Team). We assessed differences in baseline characteristics describing qualifying admissions across 3 groups: EHR-confirmed sepsis, COVID-19 sepsis, and nonsepsis status. We used a χ^2^ test of independence for categorical variables and a Kruskal-Wallis test for nonparametric continuous variables for between-group comparisons. We reported standard classification metrics (sensitivity, specificity, false positives, and false negatives) for each criterion and assessed pairwise differences between each criterion and each PSS threshold using the McNemar test. We reported the balanced accuracy and the diagnostic odds ratio^40^ as measures of classification independent of prevalence, given the low sepsis prevalence in the study. We did not report the area under the curve or calibration because SIRS, SOFA, and qSOFA have a single threshold prespecified by the literature, resulting in an inability to compare these metrics with the SPM. To quantify timeliness of each criterion’s threshold with respect to time zero, we reported the median time to the first threshold score during EHR-confirmed sepsis admissions for which a threshold was met with negative and positive values indicating that the threshold was reached before or after time zero, respectively. To better understand the timing between the instant the threshold was first met and time zero beyond simple summary statistics, we used an admission-level longitudinal plot to visualize how long after time zero a threshold was met. We used a PSS of 8 or greater as a representative threshold given the vendor recommendation for PSS threshold as a score of 5 to 8.^31^ However, we included all PSS thresholds between 5 and 10 in eFigure 1 and eTables 2 and 4 in Supplement 1.

## Results

**Figure 1.**
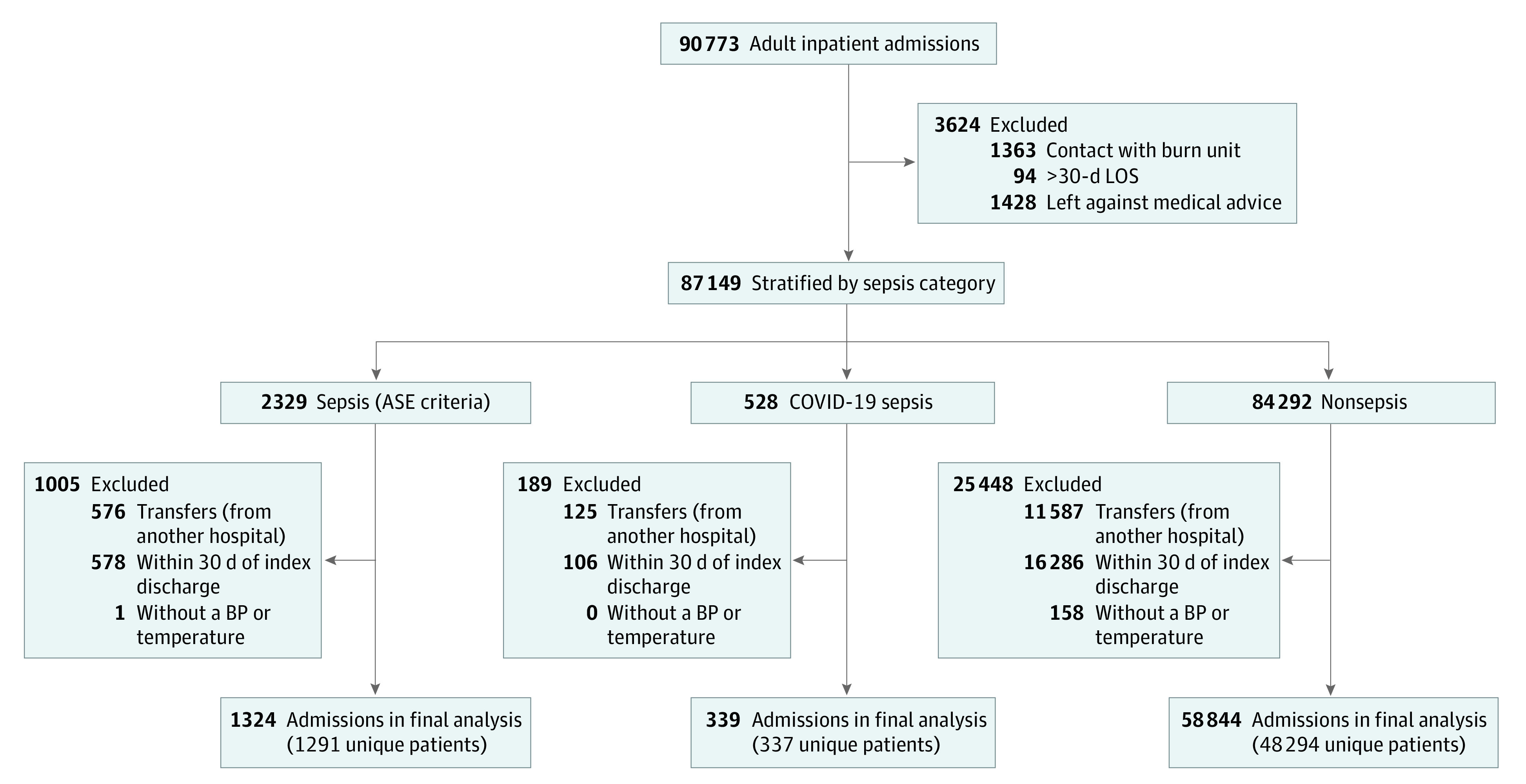
Flow Diagram of Qualifying Admissions by Sepsis Category ASE indicates Adult Sepsis Event; BP, blood pressure; LOS, length of stay.

Of 90 773 adult admissions recorded from June 5, 2019, through December 31, 2020, our analytic data set included 60 507 admissions, comprising 49 369 unique patients, that met the inclusion criteria (Figure 1). Baseline characteristics that describe qualifying admissions are provided across the 3 groups: EHR-confirmed sepsis, COVID-19 sepsis, and nonsepsis status (Table 1). There were 1324 patients with EHR-confirmed sepsis (median [IQR] age, 63 [51-73] years; 699 [52.8%] male patients; 298 [22.5%] Black or African American non-Hispanic/Latinx patients; 46 [3.5%] Hispanic/Latinx patients; 945 [71.4%] White non-Hispanic/Latinx patients); 339 patients with COVID-19 sepsis (median [IQR] age, 69 [60-77] years; 183 [54.0%] male patients; 98 [28.9%] Black or African American non-Hispanic/Latinx patients; 36 [10.6%] Hispanic/Latinx patients; and 189 [55.8%] White non-Hispanic/Latinx patients), and 58 884 nonsepsis admission (median [IQR] age, 60 [42-72] years; 26 632 [45.2%] male patients; 12 698 [21.6%] Black or African American non-Hispanic Latinx patients; 3367 [5.7%] Hispanic/Latinx patients; 40 491 White non-Hispanic Latinx patients). Overall, 1663 admissions (2.7%; 95% CI, 2.6%-2.9%) met sepsis criteria, 339 (20.4%; 95% CI, 18.4%-22.3%) of which were diagnosed with COVID-19. Those with EHR-confirmed sepsis were older and more likely to be classified as White compared with all-cause admissions. Immunocompromised status, atherosclerotic cardiovascular disease, liver disease, and chronic obstructive pulmonary disease were more prevalent conditions among sepsis admissions compared with all other groups. Sepsis admissions also had a longer length of stay, a greater proportion of intensive care unit (ICU) utilization, and higher rates of mortality compared with nonsepsis admissions. Those with COVID-19 sepsis were notably older; had a higher body mass index; comprised a larger proportion of males, Black, and Latinx patients; and had a higher proportion with diabetes compared with the other groups. COVID-19 sepsis admissions also had the highest proportion of initial contact in the ED, ICU utilization, longer length of stay, and substantially higher in-hospital and composite 30-day mortality.

**Table 1.** Baseline Characteristics of Inpatient Admissions by Sepsis Category^a^

Characteristic	Admissions, No. (%)		
	ASE sepsis (n = 1324)	COVID-19 sepsis (n = 339)	Nonsepsis (n = 58 844)
Age, median (IQR), y	63.00 (51.00-73.00)	69.00 (60.00-77.00)	60.00 (42.00-72.00)
Sex			
Female	625 (47.2)	155 (45.7)	32 200 (54.7)
Male	699 (52.8)	183 (54.0)	26 621 (45.2)
Race and ethnicity			
Black or African American	298 (22.5)	98 (28.9)	12 698 (21.6)
Hispanic/Latinx	46 (3.5)	36 (10.6)	3367 (5.7)
White non-Hispanic/Latinx	945 (71.4)	189 (55.8)	40 491 (68.8)
Other^b^	33 (2.5)	15 (4.4)	2195 (3.7)
Missing or unknown	2 (0.2)	1 (0.3)	93 (0.2)
Body mass index^c^			
Median (IQR)	28.13 (23.47-33.93)	30.07 (25.67-37.66)	28.58 (24.14-34.03)
≥30^d^	539 (40.7)	169 (49.9)	24 660 (41.9)
<30	34 660 (57.3)	781 (59.0)	166 (49.0)
Charlson Comorbidity Index score			
0-1	352 (26.6)	121 (35.7)	27 731 (47.1)
2-5	581 (43.9)	150 (44.2)	19 855 (33.7)
>6	391 (29.5)	68 (20.1)	11 258 (19.1)
Comorbid conditions			
Immunocompromised^e^	133 (10.0)	13 (3.8)	3666 (6.2)
Malignant neoplasm, excluding skin	303 (22.9)	41 (12.1)	9726 (16.5)
Atherosclerotic cardiovascular disease	723 (54.6)	152 (44.8)	23 870 (40.6)
Liver disease	274 (20.7)	46 (13.6)	7010 (11.9)
Kidney disease	373 (28.2)	97 (28.6)	11624 (19.8)
Chronic obstructive pulmonary disease	484 (36.6)	110 (32.4)	16 902 (28.7)
Diabetes	461 (34.8)	146 (43.1)	16 387 (27.8)
Hospital location			
Tertiary medical center	938 (70.8)	139 (41.0)	31 294 (53.2)
Community-based hospital	386 (29.2)	200 (59.0)	27 550 (46.8)
ED admission source	1090 (79.4)	333 (90.5)	36 589 (60.6)
Any ICU stay	910 (68.7)	235 (69.3)	9525 (16.2)
Length of stay, median (IQR), d	9.19 (6.09-15.24)	10.05 (5.98-16.74)	3.19 (2.10-5.14)
Mortality			
In-hospital mortality	148 (11.2)	119 (35.1)	860 (1.5)
Composite in-hospital and 30-d mortality	239 (18.1)	138 (40.7)	2360 (4.0)

Abbreviations: ASE, Adult Sepsis Event; ED, emergency department; ICU, intensive care unit.

^a^ χ^2^ test was conducted for categorical variables. Kruskal-Wallis test was implemented for continuous, skewed variables.

^b^ Other is inclusive of categories in the electronic health record labeled as American Indian or Alaska Native, Asian, multiracial, Native Hawaiian or Other Pacific Islander, and Other (not further specified).

^c^ Body mass index is calculated as weight in kilograms divided by height in meters squared.

^d^ The *P* value for body mass of 30 or greater was .005, and *P *values for all other variables were <.001.

^e^ Immunocompromised indicates patient with HIV/AIDS or metastatic cancer.

We observed an inverse association between an increase in PSS threshold and sensitivity and a positive association for higher PSS threshold and specificity (Table 2). For the entire length of admissions, SIRS, SOFA, and qSOFA statistically differed in classification compared with the SPM at all PSS thresholds. SOFA criteria had the highest sensitivity at 0.97 (95% CI, 0.97-0.98) but also yielded the largest percentage of false positives at 0.57 (95% CI, 0.56-0.57) compared with all but SIRS. Within the vendor-recommended range of 5 to 8, a PSS of 8 or greater had the highest balanced accuracy for classifying a sepsis admission at 0.79 (95% CI, 0.78 to 0.80) (Table 2). SOFA criteria had the highest diagnostic odds ratio among the scores (Table 2; eFigure 1 in Supplement 1). Classification metrics for septic shock and a composite of inpatient and 30-day mortality were also analyzed (eTable 2 in Supplement 1).

**Table 2.** Performance Metrics for Classification of Admission (All Sepsis vs Nonsepsis)

All sepsis classification (n = 1663)	Performance metric (95% CI)						
	Accuracy	Sensitivity	Specificity	False negative^a^	False positive^b^	Balanced accuracy^c^	Diagnostic odds ratio^d^
PSS ≥5	0.54 (0.54-0.55)	0.95 (0.93-0.96)	0.53 (0.53-0.54)	0.05 (0.04-0.07)	0.47 (0.46-0.47)	0.74 (0.73-0.74)	19.82 (16.01-24.52)
PSS ≥6	0.63 (0.62-0.63)	0.92 (0.91-0.93)	0.62 (0.61-0.62)	0.08 (0.07-0.09)	0.38 (0.38-0.39)	0.77 (0.76-0.78)	18.63 (15.59-22.26)
PSS ≥7	0.69 (0.69-0.69)	0.89 (0.87-0.90)	0.68 (0.68-0.69)	0.11 (0.10-0.13)	0.32 (0.31-0.32)	0.79 (0.78-0.79)	17.44 (14.94-20.34)
PSS ≥8	0.74 (0.73-0.74)	0.85 (0.83-0.87)	0.73 (0.73-0.74)	0.15 (0.13-0.17)	0.27 (0.26-0.27)	0.79 (0.78-0.80)	15.96 (13.92-18.30)
PSS ≥9	0.78 (0.77-0.78)	0.82 (0.80-0.84)	0.77 (0.77-0.78)	0.18 (0.16-0.20)	0.23 (0.22-0.23)	0.80 (0.79-0.81)	15.78 (13.90-17.91)
PSS ≥10	0.81 (0.80-0.81)	0.78 (0.76-0.80)	0.81 (0.80-0.81)	0.22 (0.20-0.24)	0.19 (0.19-0.20)	0.80 (0.79-0.81)	15.10 (13.41-16.99)
SIRS	0.43 (0.43-0.44)	0.95 (0.94-0.96)	0.42 (0.41-0.42)	0.05 (0.04-0.06)	0.58 (0.58-0.59)	0.68 (0.68-0.69)	13.49 (10.82-16.81)
qSOFA	0.70 (0.69-0.70)	0.83 (0.81-0.85)	0.69 (0.69-0.70)	0.17 (0.15-0.19)	0.31 (0.30-0.31)	0.76 (0.75-0.77)	11.08 (9.73-12.61)
SOFA	0.45 (0.44-0.45)	0.97 (0.97-0.98)	0.43 (0.43-0.44)	0.03 (0.02-0.03)	0.57 (0.56-0.57)	0.70 (0.70-0.71)	28.60 (21.12-38.73)

Abbreviations: PSS, Predicting Sepsis Score; qSOFA, quick Sepsis-Related Organ Failure Assessment; SIRS, Systemic Inflammatory Response Syndrome; SOFA, Sequential Organ Failure Assessment.

^a^ False negative indicates the proportion of missed true sepsis cases.

^b^ False positive indicates the proportion of nonsepsis cases falsely classified as sepsis.

^c^ Balanced accuracy is calculated as sensitivity plus specificity divided by 2.

^d^ Diagnostic odds ratio is calculated as the positive likelihood ratio divided by the negative likelihood ratio.

**Figure 2.**
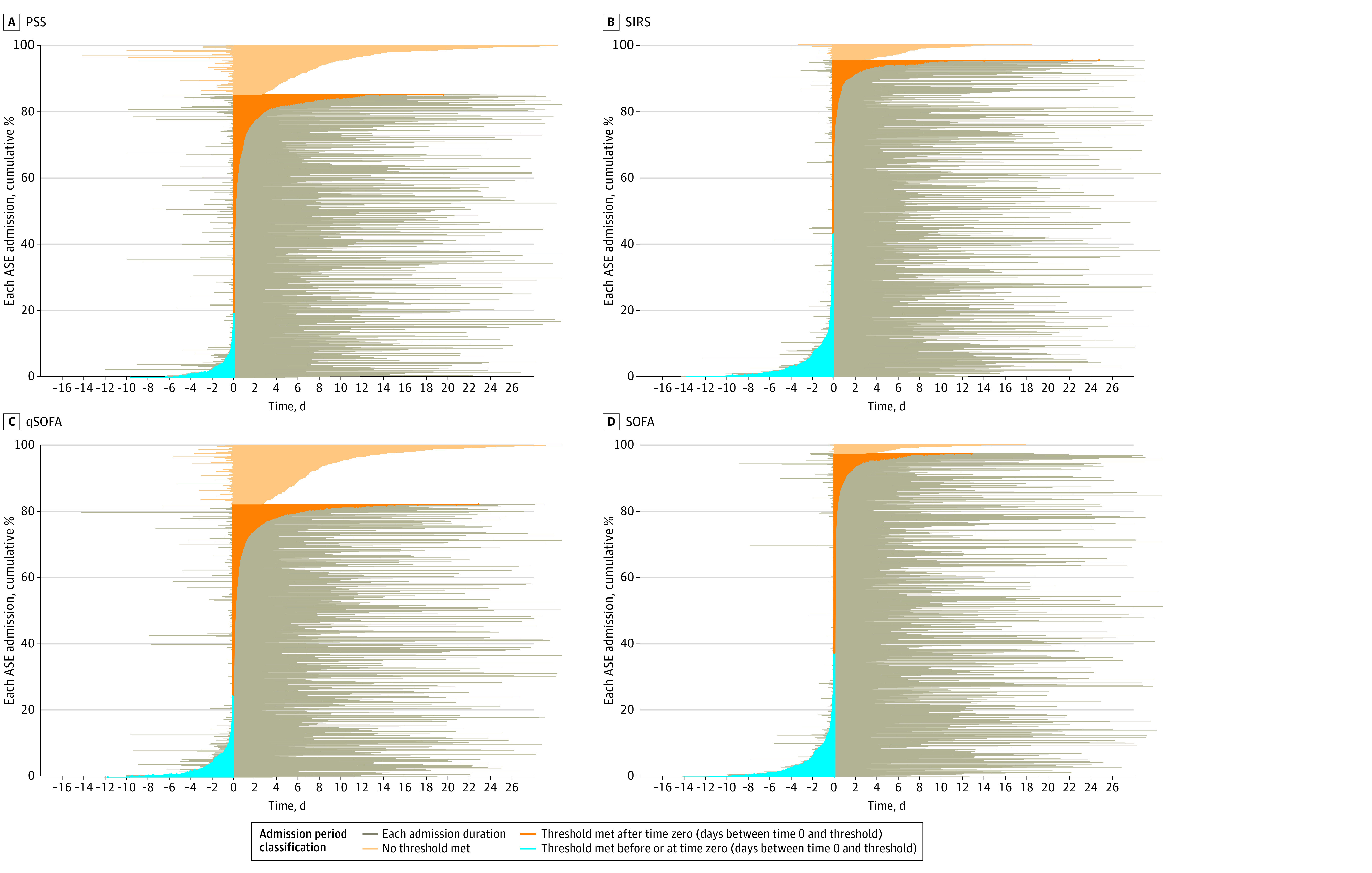
Plot of Criteria Time With Respect to Time Zero for Each Adult Sepsis Event (ASE) Admission, by Sepsis Model This figure demonstrates the difference between time zero and time of sepsis threshold positivity. The y-axis indicates the cumulative proportion of admissions, and the x-axis shows the duration of the admission in days. The point 0 on the x-axis is time zero. Those admissions with a threshold score prior to time zero are negative and are shaded in blue. Those admissions with a threshold score after time zero are positive and are shaded in orange. Those admissions without a threshold score are shaded in light orange. Gray bars along the x-axis represent the duration of each individual admission. Time zero is 15 minutes before clinician action (blood culture or antimicrobial order). PSS indicates predicting sepsis score; SIRS, Systemic Inflammatory Response Syndrome; SOFA, Sequential Organ Failure Assessments; qSOFA, quick Sepsis-Related Organ Failure Assessment.

All the prediction scores had a median time to threshold after time zero (Table 3). SIRS criteria were positive in the largest proportion of EHR-confirmed sepsis admissions before time zero (573 [43.3%]). Median (IQR) time to SIRS threshold was 7.00 (IQR, −105.00 to 108.00) minutes after time zero. Comparatively, the SPM at a threshold of PSS 8 or greater was positive in only 261 EHR-confirmed sepsis admissions (19.7%) before time zero and reached threshold a median (IQR) of 68.00 (6.75-605.75) minutes after time zero. The SPM and qSOFA missed a larger proportion of cases than SIRS and SOFA due to no threshold being met (eFigure 2 in Supplement 1) or the threshold being met long after time zero (Figure 2). When comparing timing of score thresholds with the time of onset of organ dysfunction by eSOFA criteria, we saw similar trends, with SIRS performing best, followed by SOFA, qSOFA, and the SPM (eTable 3 in Supplement 1). Results pertaining to additional PSS thresholds can be found in eFigure 3 and eTable 4 in Supplement 1.

**Table 3.** Performance Metrics for Admissions With Respect to Time Zero Among 1324 Electronic Health Record–Confirmed Sepsis Admissions^a^

Performance metric	Diagnostic criteria, No. (%)			
	PSS	SIRS	qSOFA	SOFA
Difference between time of threshold and time zero, median (IQR), min^b^	68.00 (6.75-605.75)	7.00 (−105.00 to 108.00)	74.00 (−22.25 to 599.25)	28.00 (−108.50 to 134.00)
Threshold score before or at time zero^c^	261 (19.7)	573 (43.3)	329 (24.8)	494 (37.3)
Threshold score after time zero^c^	867 (65.5)	688 (52.0)	759 (57.3)	794 (60.0)
Threshold score not met	196 (14.8)	63 (4.8)	236 (17.8)	36 (2.7)

Abbreviations: PSS, Predicting Sepsis Score; qSOFA, quick Sepsis-Related Organ Failure Assessment; SIRS; Systemic Inflammatory Response Syndrome; SOFA, Sequential Organ Failure Assessment.

^a^ Sepsis is defined as meeting Adult Sepsis Event criteria.

^b^ Time zero indicates 15 minutes before clinician action (blood culture or antimicrobial order). Positive and negative values indicate threshold being met before and after time zero, respectively. Difference calculated only for ASE admissions where a threshold was met.

^c^ Threshold score for PSS was 8 or greater; SIRS, 2 or greater; qSOFA, 2 or greater; SOFA, 2 or greater.

## Discussion

Although the SPM demonstrated better balanced accuracy and specificity for sepsis at higher-threshold PSS (8 to 10), it also missed a higher proportion of true cases and was far less timely in comparison with SIRS and SOFA. Initial clinician action indicating suspicion for infection (antimicrobial or blood culture order) occurred a median time of 68 to 145 minutes prior to threshold score when using higher, more accurate PSS thresholds between 8 and 10. Poor timeliness combined with increased score complexity and lack of transparency of the SPM epitomizes its major flaw: it appears to predict sepsis long after the clinician has recognized possible sepsis and acted on that suspicion. This is consistent with prior research,^31^ demonstrating a lack of plausible clinical benefit of the SPM.

At higher PSS scores (8 to 10), only 12.9% to 19.7% of patients could have been identified by the SPM in a clinically relevant time prior to clinician action (Table 3). In addition to poor timeliness of the SPM, there was also a high proportion of patients at higher PSS thresholds with confirmed sepsis who never reached a threshold score, 14.8% to 21.5% for scores of 8 to 10. Although setting higher PSS thresholds decreased false-positive rates, it also resulted in higher than acceptable false-negative rates and amplified problems with timeliness of detection. These findings suggest that the SPM has limited potential to shorten time to clinician action compared with alternative criteria.

Given the existing observational data regarding the importance of early antimicrobial administration, the patients who would benefit most from early recognition, resource allocation, and appropriate and timely therapeutic intervention are those at the highest risk for a poor outcome. Development of a prediction tool that accurately captures this high-risk group in a timely manner should be the focus of future model development. Epic has subsequently revamped its sepsis algorithm to version 2.0 in response to critical evaluation of the algorithm in a clinical setting.^41^ It is yet to be determined whether updating the sepsis definition in the new model will address any of the existing limitations to timeliness that may be due to other aspects of model development in order to meaningfully impact sepsis recognition or management.

### Strengths and Limitations

The strengths of our study include a large sample size and inclusion of patients from both community-based and tertiary referral hospitals. Utilization of the CDC ASE criteria as well as separately defining COVID-19–associated sepsis rather than *ICD *coding also represents a significant strength in defining a reference-standard diagnosis that is not subject to variations in documentation and coding practices across institutions. Concurrent analysis of SIRS, qSOFA, and SOFA scores provides comparative data to support conclusions about the clinical utility of the SPM. Capture of time-specific events including clinician action and time of organ dysfunction in comparison with time of score threshold positivity permits conclusions not only about the validity of the scores but also the potential clinical utility to improve timely interventions for early sepsis. To our knowledge, no prior studies have assessed the SPM as it relates to both validity and timeliness in sepsis prediction compared with existing models.

This study also has limitations, including investigation of only a single health system, the observational study design, the inherent heterogeneity of sepsis, reliance on EHR data, and missing data. Because the SPM is available during all phases of in-hospital acute care, both community- and hospital-onset sepsis were included. While community- and hospital-onset sepsis might be distinct entities with unique phenotypes,^42,43,44,45,46^ the SPM does not differentiate, and, as such, we felt that it was vital to include both entities in the study. Additionally, this study was undertaken during the COVID-19 pandemic. Although the SPM was derived prior to the pandemic, we felt that COVID-19 was important to include, as it represented a primary source of sepsis during the study period. The generalizability of the study findings is limited by the single health system setting; however, we feel that minimal exclusion criteria and large sample size mitigates those concerns.

## Conclusions

In this cohort study of 60 507 hospital admissions, we found that although the SPM marginally outperformed existing prediction scores in balanced accuracy for classification of sepsis, it suffers from poor timeliness, limiting its clinical application for sepsis diagnosis and treatment. As with all questions of testing performance, the balance between missed true cases and overtreatment of false positives must be weighed. In the case of sepsis, prioritization of timely treatment is paramount, given the potentially severe consequences when the diagnosis is missed or delayed. Irrespective of the clinical utility of the SPM as a prediction model, sepsis remains an area that is underresearched and underdeveloped from the perspective of prediction modeling. The current tools continue to leave enormous gaps in our ability to fully determine which patients need urgent treatment for sepsis and reduce the high burden of associated negative health outcomes.

## Notes

Supplement 1. **eFigure 1.** Diagnostic Odds Ratios for Sepsis, by Model **eFigure 2.** Kaplan-Meier Analysis of Time to Threshold Positivity vs Time Zero **eFigure 3.** Plot of Criteria Time With Respect to Time Zero for Each EHR-Confirmed Admission, PSS Only **eTable 1.** Missing Data Stratified by Admission Type **eTable 2.** Additional Performance Metrics for Classification of Admission **eTable 3.** Performance Metrics for Admissions With Respect to Organ Dysfunction Among 1324 EHR-Confirmed Admissions **eTable 4.** Performance Metrics for Admissions With Respect to Time Zero for 1324 EHR-Confirmed Admissions, PSS Only

Supplement 2.
Data Sharing Statement
