# Supplementary material for: Sepsis Prediction Model for Determining Sepsis vs SIRS, qSOFA, and SOFA
Source: JAMA Netw Open. 2023 Aug 25;6(8):e2329729. doi: 10.1001/jamanetworkopen.2023.29729 (PMC10457723; doi:10.1001/jamanetworkopen.2023.29729)
Supplement: Supplement 1. — eFigure 1. Diagnostic Odds Ratios for Sepsis, by Model eFigure 2. Kaplan-Meier Analysis of Time to Threshold Positivity vs Time Zero eFigure 3. Plot of Criteria Time With Respect to Time Zero for Each EHR-Confirmed Admission, PSS Only eTable 1. Missing Data Stratified by Admission Type eTable 2. Additional Performance Metrics for Classification of Admission eTable 3. Performance Metrics for Admissions With Respect to Organ Dysfunction Among 1324 EHR-Confirmed Admissions eTable 4. Performance Metrics for Admissions With Respect to Time Zero for 1324 EHR-Confirmed Admissions, PSS Only [file jamanetwopen-e2329729-s001.pdf]

Supplemental Online Content

Schertz AR, Lenoir KM, Bertoni AG, Levine BJ, Mongraw-Chaffin M, Thomas KW. Sepsis Prediction Model for determining sepsis vs SIRS, qSOFA, and SOFA. *JAMA Netw Open.* 2023;6(8):e2329729. doi:10.1001/jamanetworkopen.2023.29729

eFigure 1. Diagnostic Odds Ratios for Sepsis, by Model

eFigure 2. Kaplan-Meier Analysis of Time to Threshold Positivity vs Time Zero

eFigure 3. Plot of Criteria Time With Respect to Time Zero for Each EHR-Confirmed Admission, PSS Only

eTable 1. Missing Data Stratified by Admission Type

eTable 2. Additional Performance Metrics for Classification of Admission

eTable 3. Performance Metrics for Admissions With Respect to Organ Dysfunction Among 1324 EHR-Confirmed Admissions

eTable 4. Performance Metrics for Admissions With Respect to Time Zero for 1324 EHR-Confirmed Admissions, PSS Only

This supplemental material has been provided by the authors to give readers additional information about their work.

© 2023 Schertz AR et al. *JAMA Network Open.*

eFigure 1. Diagnostic Odds Ratios for Sepsis, by Model

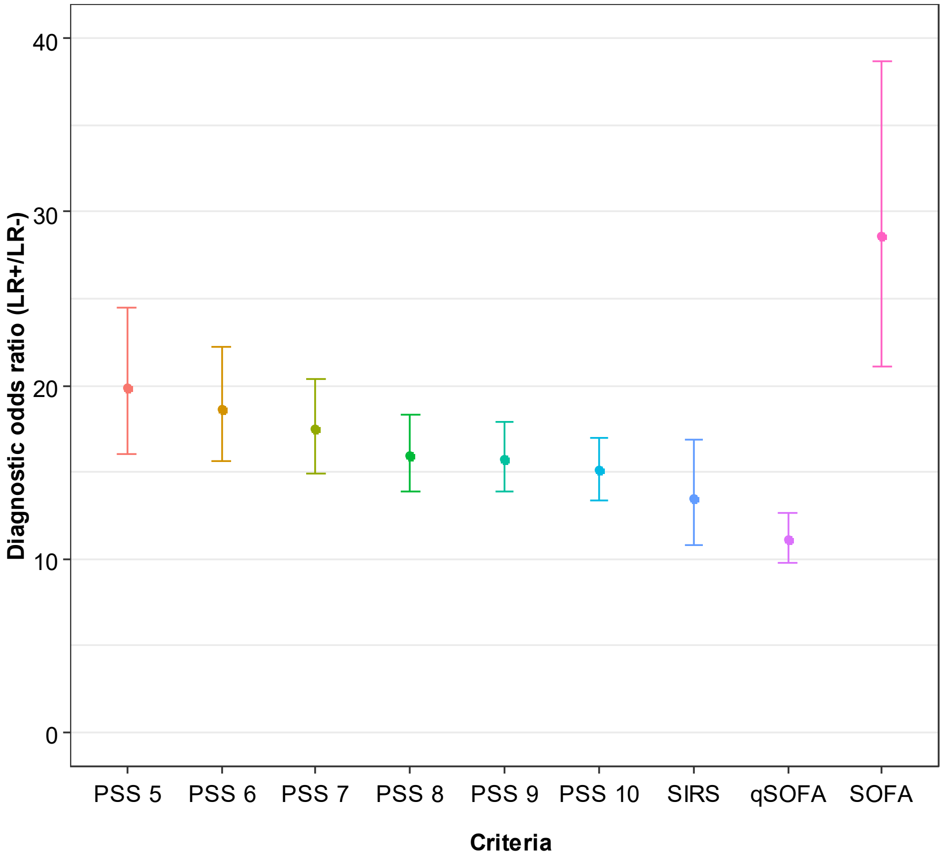

Figure demonstrates the diagnostic odds ratio for all analyzed PSS, SIRS, qSOFA and SOFA. Sepsis includes both electronic health record (EHR)-confirmed and COVID-19 sepsis. Error bars show 95% confidence intervals. Note: Diagnostic odds ratio = Positive likelihood ratio / negative likelihood ratio, with higher values representing better classification.

© 2023 Schertz AR et al. *JAMA Network Open.*

eFigure 2. Kaplan-Meier Analysis of Time to Threshold Positivity vs Time Zero

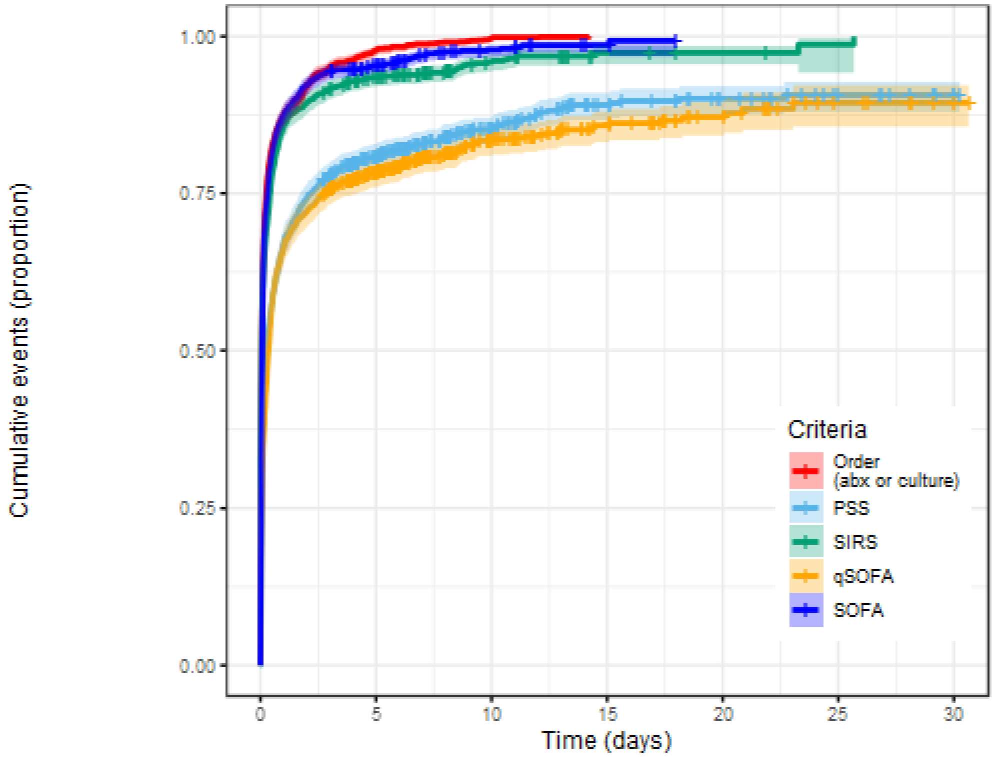

Number at risk

Order (abx or culture)	1324	29	4	0	0	0	0
PSS	1324	209	89	36	22	10	1
SIRS	1324	74	22	5	3	1	0
qSOFA	1324	238	84	41	20	7	1
SOFA	1324	53	13	3	0	0	0

Figure demonstrates the cumulative proportion of EHR-confirmed sepsis admissions meeting each sepsis criteria over a maximum admission duration of 30 days. Note: PSS threshold >8.

© 2023 Schertz AR et al. *JAMA Network Open.*

eFigure 3. Plot of Criteria Time With Respect to Time Zero for Each EHR-Confirmed Admission, PSS Only

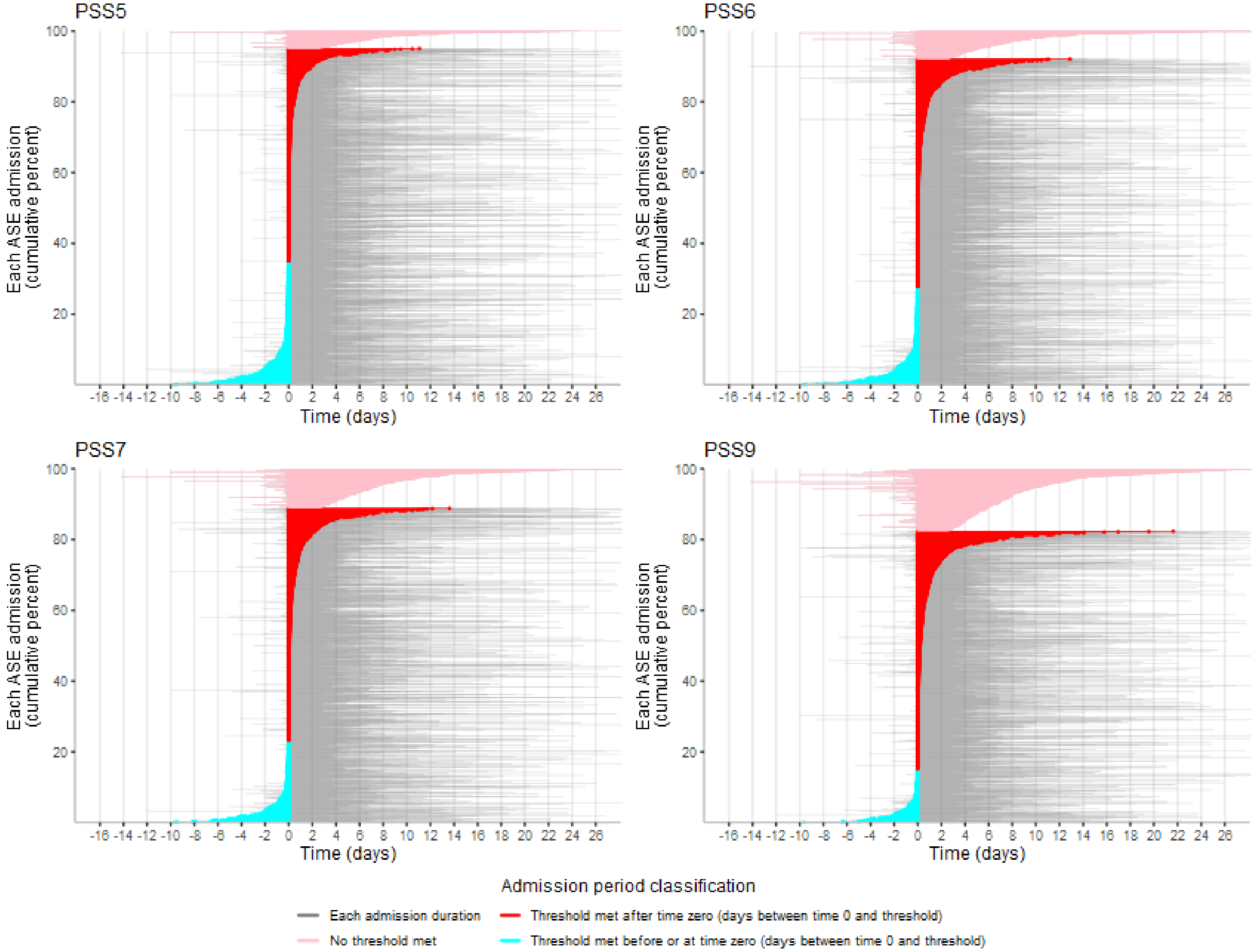

Figure demonstrates the difference between time zero and time of sepsis threshold positivity. The Y-axis indicates the cumulative proportion of admissions, and the X-axis shows the duration of the admission in days. The point "0" on the X-axis is time zero. Those admissions with a threshold score prior to time zero are negative and are shaded in light blue. Those admissions with a threshold score after time zero are positive and are shaded in red. Those admissions without a threshold score are shaded in pink. Gray bars along the X-axis represent the duration of each individual admission. Note: Time zero is 15 minutes before provider action (blood culture or antimicrobial order).

© 2023 Schertz AR et al. *JAMA Network Open.*

eTable 1. Missing Data Stratified by Admission Type

Characteristic	ASE sepsis	COVID-19 sepsis	Non-sepsis
Admissions, No.	1,324	339	58,844
Temperature	1324 (100.0)	339 (100.0)	58844 (100.0)
Systolic blood pressure	1324 (100.0)	339 (100.0)	58844 (100.0)
Heart rate	1324 (100.0)	339 (100.0)	58844 (100.0)
Respiration rate	1324 (100.0)	339 (100.0)	58842 (100.0)
Creatinine	1323 (99.9)	339 (100.0)	51228 (87.1)
Bilirubin	1277 (96.5)	333 (98.2)	38464 (65.4)
Lactate	1179 (89.0)	299 (88.2)	19648 (33.4)
Glasgow coma scale	1324 (100.0)	339 (100.0)	56847 (96.6)
Platelets	1324 (100.0)	339 (100.0)	55657 (94.6)
White blood cell count	1324 (100.0)	339 (100.0)	55682 (94.6)

These measures contribute to the calculation of SIRS, qSOFA, and SOFA, but do not need to all be complete within each criteria calculation in order to reach the positive threshold.

eTable 2. Additional Performance Metrics for Classification of Admission

Classification Metric	Accuracy	Sensitivity	Specificity	False negative[a]	False positive[b]	Balanced accuracy[c]	Diagnostic odds ratio[d]
**Septic shock (n=839)**							
PSS ≥5	0.53 (0.53-0.54)	0.98 (0.96-0.99)	0.53 (0.52-0.53)	0.02 (0.01-0.04)	0.47 (0.47-0.48)	0.75 (0.75-0.76)	45.29 (29.06-70.60)
PSS ≥6	0.62 (0.61-0.62)	0.95 (0.94-0.97)	0.61 (0.61-0.62)	0.05 (0.03-0.06)	0.39 (0.38-0.39)	0.78 (0.78-0.79)	32.25 (23.38-44.50)
PSS ≥7	0.68 (0.68-0.68)	0.94 (0.92-0.96)	0.68 (0.67-0.68)	0.06 (0.04-0.08)	0.32 (0.32-0.33)	0.81 (0.80-0.82)	33.83 (25.34-45.17)
PSS ≥8	0.73 (0.73-0.73)	0.91 (0.89-0.93)	0.73 (0.72-0.73)	0.09 (0.07-0.11)	0.27 (0.27-0.28)	0.82 (0.81-0.83)	28.31 (22.22-36.07)
PSS ≥9	0.77 (0.77-0.77)	0.89 (0.87-0.91)	0.77 (0.76-0.77)	0.11 (0.09-0.13)	0.23 (0.23-0.24)	0.83 (0.82-0.84)	27.75 (22.26-34.60)
PSS ≥10	0.80 (0.80-0.80)	0.86 (0.84-0.89)	0.80 (0.80-0.80)	0.14 (0.11-0.16)	0.20 (0.20-0.20)	0.83 (0.82-0.84)	25.12 (20.61-30.61)
SIRS	0.42 (0.42-0.43)	0.99 (0.98-0.99)	0.41 (0.41-0.42)	0.01 (0.01-0.02)	0.59 (0.58-0.59)	0.70 (0.70-0.70)	48.53 (27.44-85.83)
qSOFA	0.69 (0.69-0.69)	0.98 (0.97-0.99)	0.69 (0.68-0.69)	0.02 (0.01-0.03)	0.31 (0.31-0.32)	0.83 (0.83-0.84)	105.87 (65.48-171.20)
SOFA	0.43 (0.43-0.44)	0.99 (0.98-1.00)	0.43 (0.42-0.43)	0.01 (0.00-0.02)	0.57 (0.57-0.58)	0.71 (0.71-0.71)	88.27 (41.95-185.77)
**30-day mortality (n= 2,756)**							
PSS ≥5	0.55 (0.55-0.56)	0.87 (0.86-0.88)	0.54 (0.53-0.54)	0.13 (0.12-0.14)	0.46 (0.46-0.47)	0.70 (0.70-0.71)	7.94 (7.09-8.90)
PSS ≥6	0.63 (0.63-0.64)	0.82 (0.81-0.84)	0.62 (0.62-0.63)	0.18 (0.16-0.19)	0.38 (0.37-0.38)	0.72 (0.72-0.73)	7.72 (6.99-8.53)
PSS ≥7	0.69 (0.69-0.70)	0.76 (0.75-0.78)	0.69 (0.69-0.69)	0.24 (0.22-0.25)	0.31 (0.31-0.31)	0.73 (0.72-0.73)	7.14 (6.53-7.81)
PSS ≥8	0.74 (0.73-0.74)	0.72 (0.70-0.73)	0.74 (0.73-0.74)	0.28 (0.27-0.30)	0.26 (0.26-0.27)	0.73 (0.72-0.74)	7.13 (6.55-7.76)
PSS ≥9	0.77 (0.77-0.78)	0.67 (0.65-0.69)	0.78 (0.78-0.78)	0.33 (0.31-0.35)	0.22 (0.22-0.22)	0.73 (0.72-0.73)	7.18 (6.62-7.80)
PSS ≥10	0.80 (0.80-0.80)	0.62 (0.60-0.64)	0.81 (0.81-0.81)	0.38 (0.36-0.40)	0.19 (0.19-0.19)	0.72 (0.71-0.72)	6.99 (6.46-7.57)
SIRS	0.44 (0.44-0.44)	0.86 (0.84-0.87)	0.42 (0.42-0.42)	0.14 (0.13-0.16)	0.58 (0.58-0.58)	0.64 (0.63-0.65)	4.40 (3.95-4.90)
qSOFA	0.70 (0.70-0.70)	0.75 (0.74-0.77)	0.70 (0.69-0.70)	0.25 (0.23-0.26)	0.30 (0.30-0.31)	0.73 (0.72-0.74)	7.11 (6.50-7.76)
SOFA	0.46 (0.45-0.46)	0.92 (0.91-0.93)	0.44 (0.43-0.44)	0.08 (0.07-0.09)	0.56 (0.56-0.57)	0.68 (0.67-0.69)	9.35 (8.12-10.77)

[a]False negative = proportion of missed true sepsis cases.
[b]False positive = proportion of non-sepsis cases falsely classified as sepsis.
[c]Diagnostic odds ratio = positive likelihood ratio / negative likelihood ratio.
ASE = Adult Sepsis Event, PSS = Predicting Sepsis Score, SIRS = Systemic Inflammatory Response Syndrome, qSOFA = quick sepsis-related Organ Failure Assessment, SOFA = Sequential Organ Failure Assessment.

© 2023 Schertz AR et al. *JAMA Network Open.*

eTable 3. Performance Metrics for Admissions With Respect to Organ Dysfunction Among 1324 EHR-Confirmed Admissions

	Diagnostic Criteria			
**Performance metric**	**PSS**	**SIRS**	**qSOFA**	**SOFA**
Difference between time of threshold and organ dysfunction[a], Minutes, Median [IQR]	-35.00 [-939.00 to 266.50]	-122.00 [-1675.00 to -16.00]	-53.50 [-791.00 to 129.00]	-67.00 [-1389.50 to 0.00]
Threshold score[b] before organ dysfunction, No. (%)	516 (39.0)	953 (72.0)	652 (49.2)	759 (57.3)
Threshold score after organ dysfunction, No. (%)	516 (39.0)	308 (23.3)	436 (32.9)	529 (40.0)
Threshold score not met, No. (%)	196 (14.8)	63 (4.8)	236 (17.8)	36 (2.7)

[a]Organ dysfunction = $\geq$1 organ dysfunction criteria optimized for EHR (eSOFA) criteria positive. Positive and negative values indicative of threshold being met before and after time of initial organ dysfunction, respectively. Difference calculated only for ASE admissions where a threshold was met.
[b]Threshold score: PSS$\geq$8, SIRS$\geq$2, qSOFA$\geq$2, SOFA$\geq$2.

© 2023 Schertz AR et al. *JAMA Network Open.*

eTable 4. Performance Metrics for Admissions With Respect to Time Zero for 1324 EHR-Confirmed Admissions, PSS Only

	Diagnostic Criteria				
**Performance metric**	**PSS 5**	**PSS 6**	**PSS 7**	**PSS 9**	**PSS 10**
**Provider action**					
Difference between time of threshold and time zero[a], Minutes, Median [IQR]	20.00 [-57.75 to 141.50]	32.00 [-18.00 to 332.50]	50.00 [-3.25 to 464.25]	93.50 [18.00-739.50]	145.00 [26.00-1007.00]
Threshold score[b] before or at time zero, No. (%)	464 (35.0)	368 (27.8)	309 (23.3)	202 (15.3)	171 (12.9)
Threshold score after time zero, No. (%)	794 (60.0)	851 (64.3)	867 (65.5)	888 (67.1)	868 (65.6)
Threshold score not met, No. (%)	66 (5.0)	105 (7.9)	148 (11.2)	234 (17.7)	285 (21.5)
**Organ dysfunction**					
Difference between time of threshold and organ dysfunction[c], Minutes, Median [IQR]	-104.00 [-1464.00 to 12.00]	-70.00 [-1209.50 to 66.00]	-53.00 [-1073.25 to 143.75]	-21.50 [-774.50 to 365.75]	-7.00 [-676.50 to 489.00]
Threshold score before or at time zero, No. (%)	884 (66.8)	768 (58.0)	685 (51.7)	559 (42.2)	497 (37.5)
Threshold score after time zero, No. (%)	374 (28.2)	451 (34.1)	491 (37.1)	531 (40.1)	542 (40.9)
Threshold score not met, No. (%)	66 (5.0)	105 (7.9)	148 (11.2)	234 (17.7)	285 (21.5)

[a]Time zero: 15 minutes before provider action (blood culture or antimicrobial order). Positive and negative values indicative of threshold being met before and after time zero, respectively. Difference calculated only for ASE admissions where a threshold was met.
[b]Threshold score: PSS ≥ enumerated threshold
[c]Organ dysfunction = ≥1 organ dysfunction criteria optimized for EHR (eSOFA) criteria positive. Positive and negative values indicative of threshold being met before and after time of initial organ dysfunction, respectively. Difference calculated only for ASE admissions where a threshold was met.

© 2023 Schertz AR et al. *JAMA Network Open.*
