# Supplementary material for: Sepsis Prediction Model for Determining Sepsis vs SIRS, qSOFA, and SOFA
Source: JAMA Netw Open. 2023 Aug 25;6(8):e2329729. doi: 10.1001/jamanetworkopen.2023.29729 (PMC10457723; doi:10.1001/jamanetworkopen.2023.29729)
Supplement: Supplement 2. — Data Sharing Statement [file jamanetwopen-e2329729-s002.pdf]

Data Sharing Statement

Schertz. Sepsis Prediction Model for Determining Sepsis vs SIRS, qSOFA, and SOFA. *JAMA Netw Open*. Published August 18, 2023. doi:10.1001/jamanetworkopen.2023.29729

Data
Data available: No

Additional Information
Explanation for why data not available: Data are available upon request with appropriate approvals.
